# Crystal Structure of *Escherichia coli* CusC, the Outer Membrane Component of a Heavy Metal Efflux Pump

**DOI:** 10.1371/journal.pone.0015610

**Published:** 2011-01-07

**Authors:** Rithika Kulathila, Ragini Kulathila, Mridhu Indic, Bert van den Berg

**Affiliations:** University of Massachusetts Medical School, Program in Molecular Medicine, Worcester, Massachusetts, United States of America; University of Cambridge, United Kingdom

## Abstract

**Background:**

While copper has essential functions as an enzymatic co-factor, excess copper ions are toxic for cells, necessitating mechanisms for regulating its levels. The *cusCBFA* operon of *E. coli* encodes a four-component efflux pump dedicated to the extrusion of Cu(I) and Ag(I) ions.

**Methodology/Principal Findings:**

We have solved the X-ray crystal structure of CusC, the outer membrane component of the Cus heavy metal efflux pump, to 2.3 Å resolution. The structure has the largest extracellular opening of any outer membrane factor (OMF) protein and suggests, for the first time, the presence of a tri-acylated N-terminal lipid anchor.

**Conclusions/Significance:**

The CusC protein does not have any obvious features that would make it specific for metal ions, suggesting that the narrow substrate specificity of the pump is provided by other components of the pump, most likely by the inner membrane component CusA.

## Introduction

Copper, while being required for the function of a number of enzymes in *Escherichia coli*, is also extremely toxic, requiring mechanisms to regulate its levels inside the cell. In *E. coli*, three systems are known that mediate tolerance to copper ions. The first of these, CopA, is an inner membrane P-type ATPase that actively pumps excess copper out of the cytoplasm [1]. The second system consists of the multicopper oxidase CueO, which may protect periplasmic enzymes from copper-mediated damage [1]. Finally, the *cus* system is responsible for copper and silver resistance in *E. coli* [1]. The *cus* system consists of two operons. The first of these, *cusRS*, encodes the histidine kinase CusS and the response regulator CusR. The second operon, *cusCFBA*, encodes the actual efflux pump. Transcription of *cusCFBA* is dependent on the concentration of copper (I) and silver (I) ions within the cell [1], [2]. Within the *cusCFBA* operon, CusA encodes an inner membrane protein belonging to the resistance-nodulation-division (RND) family. Phylogenetic analysis shows that CusA belongs to a subgroup of RND proteins involved in heavy metal extrusion [3]. CusB is a membrane fusion/adaptor protein (MFP) and CusC is an outer membrane (OM) protein belonging to the outer membrane factor (OMF family) [4]. Together, CusABC likely forms a protein complex spanning the inner membrane, periplasmic space and the outer membrane, analogous to the well-studied AcrAB-TolC multidrug efflux pump from *E. coli* and the MexAB-OprM multidrug efflux pump from *Pseudomonas aeruginosa*. CusF is a small periplasmic protein that binds copper(I) and silver(I) and may subsequently transport these ions to CusB [5]–[7]. Thus, the CusCFBA proteins form a tetrapartite system dedicated to the efflux of heavy metal ions from *E. coli*.

Much is known about the multidrug efflux pumps, exemplified by the AcrAB-TolC system from *E. coli*. Crystal structures are available for all three components individually. AcrB is an inner membrane proton antiporter and forms a trimer with large periplasmic domains [8]. The hydrophobic substrates are acquired laterally from the membrane; co-crystal structures indicate that the substrates bind within large pockets of AcrB, and variation in the structural states within the individual monomers likely reflects a cyclical rotary pump mechanism [9], [10]. The structure of the MFP AcrA is incomplete [11]; however, MexA, a closely related *P. aeruginosa* ortholog of AcrA (58% sequence identity), shows an extended structure with four domains that can likely adopt multiple conformations [12]. Structures of the OMF TolC show a striking, cannon-shaped molecule of ∼135 Å in length, consisting of an unusual α/β-barrel [13]. The protein is anchored into the OM via the 12-stranded β-barrel, with three monomers contributing four strands each. The α-barrel tapers to an almost closed conformation at the periplasmic end, necessitating a mechanism for channel opening to allow passage of the substrates, which range from bile salts and various antibiotics to secreted proteins [4]. Clues for the mechanism of channel opening have been obtained from structural studies of mutant TolC proteins [14]. Very recently, a model for the complete, assembled AcrAB-TolC pump was proposed based on crystal structures of the individual components and cysteine cross-linking data [12]. Within this model, there are only limited interactions between the AcrB inner membrane transporter and the TolC outer membrane channel. Rather, the fusion/adaptor protein AcrA is proposed to play a central role in the active pump assembly. It likely interacts with TolC and AcrB, thereby cementing the AcrB-TolC interactions and stabilizing the open state of TolC within the assembled pump [12].

For the Cus heavy atom efflux pump, less data are available. Regarding structural information, X-ray crystal structures were recently solved for CusF and the adaptor/fusion protein CusB [6], [15]. In CusF, Cu(I) and Ag(I) ions are bound in a novel metal recognition site involving two methionine residues and a tryptophan [6], [16]. The recently solved CusB membrane fusion protein structure is very similar to AcrA and MexA for three of the four domains. The fourth domain, which likely interacts with the OM channel CusC, consists of a novel, three-helix bundle [15]. Interestingly, co-crystal structures reveal several binding sites for Cu(I) and Ag(I) ions that look similar to those observed in CusF [6], with a methionine and an aromatic residue involved in metal coordination. These structures are the first of an adaptor protein in complex with a ligand, and suggest that the adaptor protein may have a more active role in metal transport, possibly by accepting substrates from CusF and delivering them to CusC [7], [15]. Very recently, the structure of the RND component of the Cus efflux pump, CusA, was reported with and without bound Cu(I) and Ag(I) ions [17]. The structure shows large differences relative to *E. coli* AcrB, explaining the previously noted differences in crystallization behavior between AcrB and CusC [18]. Binding of substrate ions induces conformational changes that suggest a pathway for ion transport [17]. The observed metal ion binding sites consist of methionine pairs/clusters, and confirm the methionine residues that were previously identified to be essential for copper resistance within the periplasmic domains of CusA [2]. Together, the metal ion binding sites delineate a transport pathway through the entire length of the protein, including the transmembrane domain. Interestingly, the structures also suggest that CusA can take up metal ions both from the cytoplasm and the periplasm [17], supporting the involvement of the periplasmic CusF protein in metal ion transport from the periplasm [6]. With the recent structure determination of CusA, the outer membrane channel CusC is the only component of the Cus efflux pump for which no structural information has been obtained so far.

While *E. coli* has seven RND efflux systems [19], the *cus* system is required for copper(I) ion resistance [1]–[3], suggesting that this system is either substrate specific or that it is the only RND system that is specifically induced in the presence of copper. Interestingly, TolC does not restore metal resistance in a *cusC* knockout [2]. Moreover, it was recently shown that the Cus pump has a narrow non-metal substrate range, distinguishing it from many other RND efflux systems [20].

Here we present the 2.3 Å X-ray crystal structure of CusC, the first structure of an outer membrane component of a heavy metal efflux pump. We have compared the CusC structure with the structures of other OMF proteins to obtain insight in whether CusC is likely to contribute to the observed substrate specificity of the Cus metal efflux pump.

## Results and Discussion

### Description of the CusC structure

**Figure 1:**
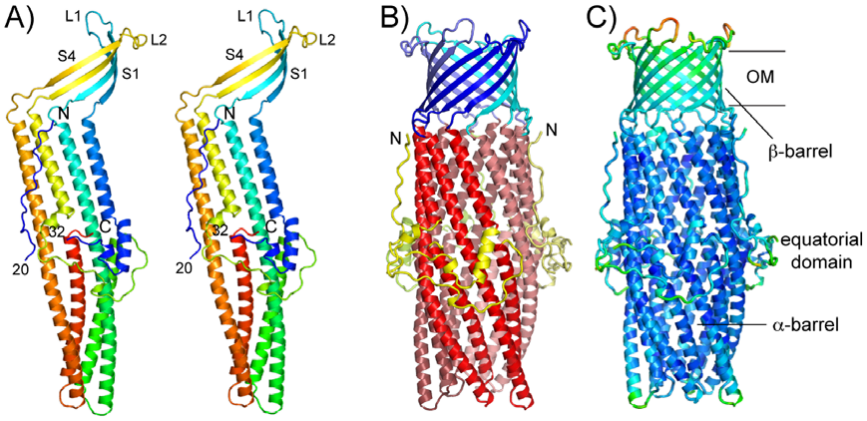
Structural overview of CusC. (A) Rainbow representation of the CusC monomer, colored from blue (N-terminus) to red (C-terminus). Selected strands (S) and extracellular loops (L) are indicated, as well as the N- and C-terminus. Residues 21–31 are not visible in the electron density, presumably because they are disordered. (B) Ribbon representation of the CusC trimer, colored by domain (blue; β-barrel, red; α-barrel, yellow; equatorial domain). The different monomers within the trimer have been indicated with different color tints. (C) Ribbon representation of the CusC trimer, colored by B-factor value (blue, low B-factors; red, high B-factors). The average B-factors for the β-barrel, α-barrel and equatorial domain are 47, 26 and 36 Å^2^ (all atoms). This and the following figures were made with PYMOL [21].

**Figure 2:**
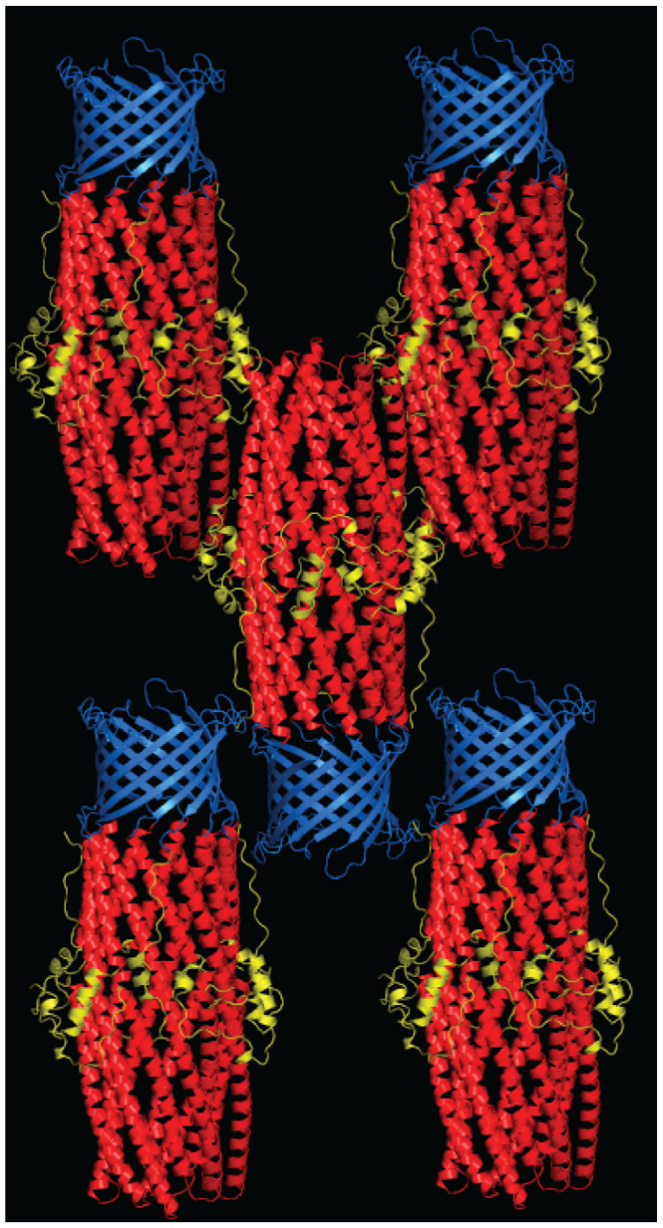
Crystal packing of CusC. Cartoon representation of the packing of CusC within the crystal, with the molecule colored by domain (β-barrel, blue; α-barrel, red; equatorial domain, yellow).

Full-length CusC (including the N-terminal cysteine residue of the mature sequence) was purified from *E. coli* membranes using dodecyl-β-maltoside (DDM). Based on its elution position on gel filtration columns, CusC forms a trimer, analogous to all other OMF proteins. CusC was crystallized by hanging drop vapor diffusion from ∼35% PEG200/20 mM Na-acetate pH 4.0/0.2 M ammonium sulphate/20 mM NaCl. The structure of CusC was solved by molecular replacement with *Pseudomonas aeruginosa* OprM, which has 45% sequence identity to CusC, as the search model (Table 1). The final CusC model consists of the complete mature sequence, with the exception of residues 21–31 within the equatorial domain, which are disordered (Fig. 1A) [21]. There is one monomer of CusC (440 residues) within the asymmetric unit, with the biological trimer formed by the symmetry within the crystal. As is the case for the other OMF proteins, CusC is a cannon-shaped molecule, forming a ∼130 Å long α/β-barrel that is composed of three monomers (Fig. 1). Each of the monomers contributes 4 β-strands to the 12-stranded β-barrel that resides within the OM, and 4 long α-helices to the periplasmic α-barrel. As with other OMF proteins, two of these helices are not continuous, with each of them formed by two shorter helices that interact end-to-end to form a long, pseudo-continuous helix. The CusC trimer forms a huge, continuous internal cavity with a cylindrical shape and a volume of ∼28,000 Å^3^ (∼90×20×20 Å), which is filled with water. Generally low B-factors indicate that the α-barrel is well-ordered, which is likely due to extensive interactions between the helical domains in the crystal lattice (Fig. 2). By contrast, the upper part of the β-barrel and the extracellular loops, as well as the equatorial domain make fewer interactions within the lattice. These parts of the protein have relatively high B-factors, indicating that these parts of CusC are intrinsically mobile (Fig. 1C and Fig. 2). The crystal packing also shows that the hydrophobic parts of the β-barrels are not closely packed, and this is the likely reason that detergent and lipid molecules are too disordered to be visible in the structure.

**Table 1:** Crystallographic parameters for CusC.

**Data Collection**	
Wavelength (Å)	1.10 (NSLS beamline X6A)
Space group	R32
Unit cell dimensions a,b,c (Å)	89.03, 89.03, 473.04
α,β,γ(°)	90, 90, 120
Resolution (Å)	50−2.2 (2.24−2.20)^1^
R_sym_ (%)	13.0 (78.0)
Completeness (%)	99.9 (99.6)
I/σ	17.3 (2.8)
Redundancy	7.4 (7.3)
Mosaicity (°)	0.29
**Refinement**	
Resolution range (Å)	20−2.3
Total no. of reflections	31287
Total no. of atoms in refinement	
Protein	3349
Sulphate	35
Water	200
Average B-factors (Å^2^)	
Protein	33
Sulphate	81
Water	38
R_work_/R_free_ 2(%)	19.8/24.3
R.m.s. deviations from ideal	
Bond lengths (Å)	0.009
Bond angles (°)	1.024
Ramachandran plot statistics (%)	
Favored/allowed/disallowed	93.6/5.9/0.5^3^

1 Values in parentheses are for the highest resolution shell.

2 R_work_ = Σ|Fo−Fc|/ΣFo. R_free_ is the cross-validation of R-factor, with 5% of the total reflections omitted in model refinement.

3 The outliers of the Ramachandran plot (Asn100, Ser311) are located in the tips of the extracellular loops, which are poorly ordered.

**Figure 3:**
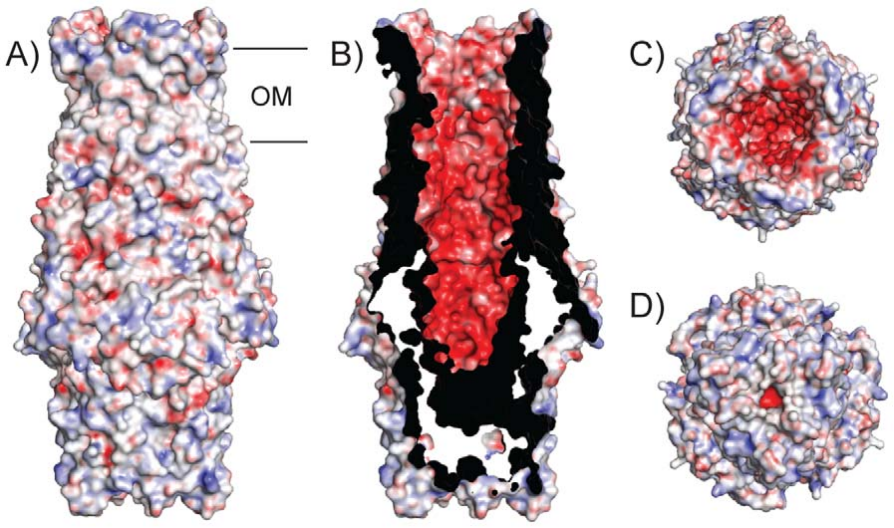
Electrostatic potentials of CusC. Surface representations of the outside (A) and inside (B) of CusC colored by charge (red; negative -40 kT/e, blue; positive +40 kT/e). The views in (C) and (D) are from the extracellular side and periplasmic side, respectively. The figures were made with the ABSP plug-in within PYMOL.

Like other OMF proteins of solved structures, CusC has an interesting charge distribution. The outside surface of the protein has no extensive positively or negatively charged patches. By contrast, the interior of the entire barrel is strikingly electronegative (Fig. 3). This feature poses the interesting question as to how monovalent cations are transported and not trapped inside the barrel. The most plausible answer to this question is that the diameter of the channel is presumably large enough (∼25 Å) for Cu(I) and Ag(I) ions to be transported together with the bulk solvent in the center of the channel. The surface of the periplasmic end of the α-barrel, which is likely to interact with CusA, is relatively hydrophobic (Fig. 3D). The channel is closed on this end by Van der Waals interactions between the Leu403 residues of the three monomers, analogous to the situation in OprM. In addition, an adjacent ring of negatively charged residues (Asp407) provides an additional layer of closure of the barrel on the periplasmic side.

### CusC is anchored by three acyl chains into the OM

**Figure 4:**
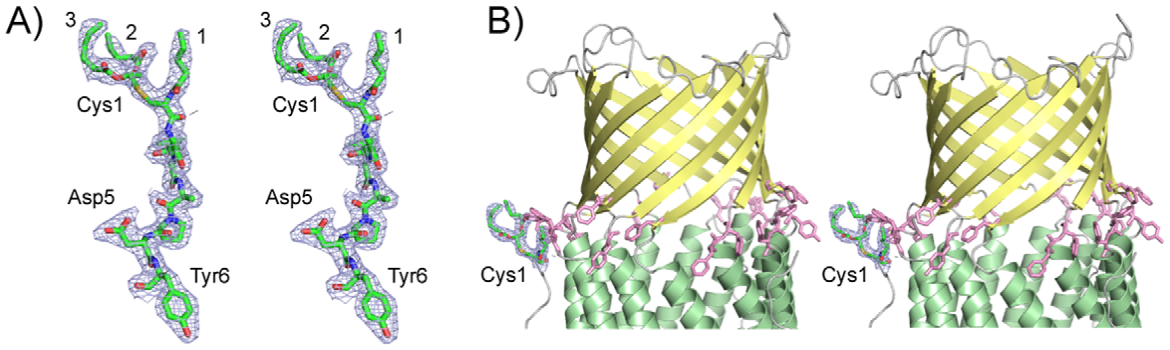
CusC is likely to be tri-acylated at the N-terminus. (A) Stereoview of the N-terminal six residues of CusC, with 2F_o_-F_c_ density shown as a blue mesh (contoured at 1.0 σ). The three acyl chains attached to Cys1 are labeled. (B) Stereoview from the side, showing the location of the lipid anchor relative to the belt of aromatic residues (purple stick models) that delineates the interface of the inner leaflet of the OM. β-strands are colored yellow, α-helices green and loops grey.

Compared to TolC, CusC as well as OprM have a N-terminal extension of about 20 residues. This segment has an interesting extended structure, which is supported by secondary structure predictions. The N-terminal extension leads from the equatorial domain to the β-barrel over a distance of ∼25 Å (Fig. 1). The N-terminal residue of this sequence (and of the entire protein) is a cysteine, likely making CusC an OM lipoprotein. In the crystal structure of OprM, the N-terminal residue of which is also a cysteine, some weak density was observed close to Cys1 that could not be modeled, but that was proposed to correspond to a (partial) palmitate residue covalently linked to the sulphur atom of Cys1 (*i.e.* with a thioester bond). In our structure of CusC, density is clearly visible for three partial acyl chains attached to Cys1 of each CusC monomer (Fig. 4A); one single chain and another chain that is clearly branched. Thus, analogous to the *E. coli* Braun lipoprotein and many other bacterial lipoproteins, CusC is most likely triacylated at the N-terminal cysteine residue with N-acyl and S-diacylglycerol functions [22]. Since there is only density for 4–5 carbon atoms of each acyl chain, the length of the acyl chains is not clear. The acyl chains are located in the same region as the aromatic belt of the inner leaflet of the OM (Fig. 4B), indicating that, as expected, they are located in the interface region of the OM. To our knowledge this is the first structural evidence for a triacylated (OM) protein. The observation that CusC is likely to be triacylated suggests that the closely similar OprM channel is modified in a similar way, and not with just a palmitoyl chain attached to the Cys1 sulphur atom, as previously proposed [23].

The reason why CusC (and possibly OprM as well) is a triacylated OM lipoprotein is not clear. Like other OM proteins, CusC and OprM have a hydrophobic region of 20–25 Å wide that serves to anchor the protein in the OM. Interestingly, CusC has an outward-facing serine residue in the center of the OM, raising the possibility that the hydrophobicity of the TM segments may not be entirely sufficient to drive Bam-assisted insertion into the OM. However, it is not clear if the somewhat polar character of the CusC OM transmembrane segment is the reason for the presence of lipid anchors, since OprM does not have a polar residue exposed to lipid. Another possible reason for the requirement of membrane-anchoring acyl chains could be the presence of very large periplasmic domains in CusC and OprM, possibly affecting the insertion of the channel into the OM as mediated by the Bam complex. However, the fact that TolC, which does not get acylated, has a very similar architecture compared to CusC/OprM strongly suggests other reasons for the presence of additional membrane-anchors in CusC and OprM.

### Comparison of CusC with TolC and other OMF proteins

**Figure 5:**
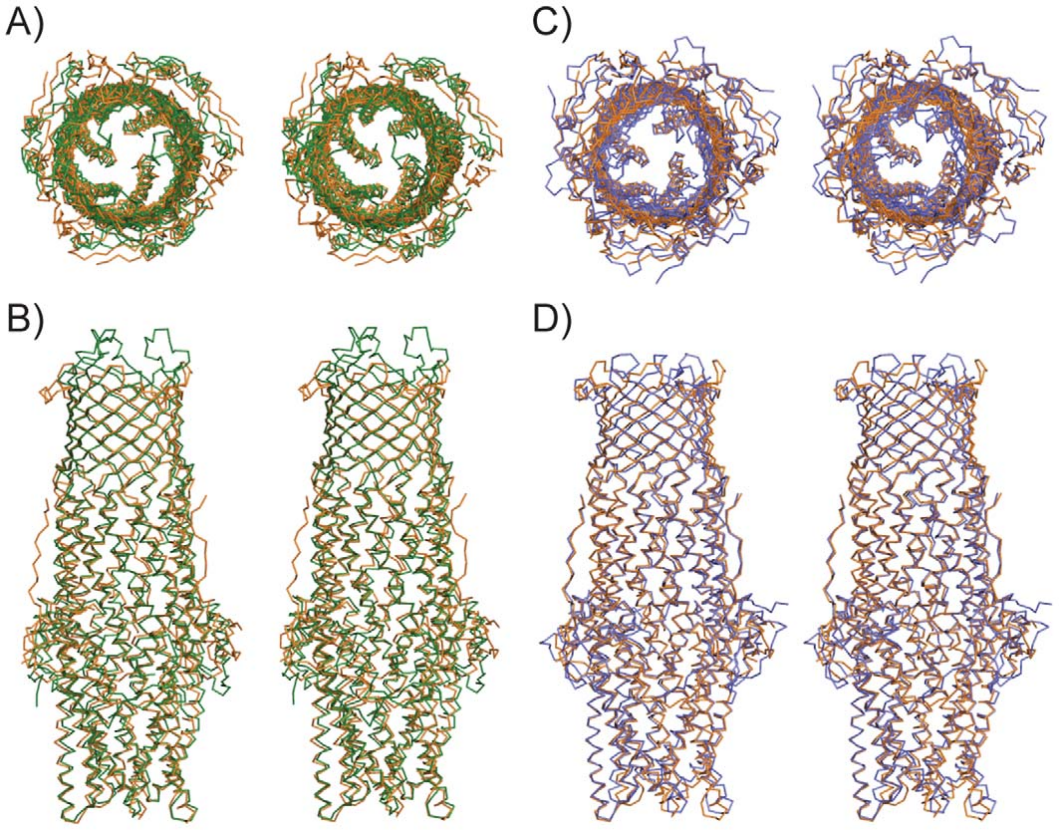
Structural comparisons between CusC, TolC and OprM. Stereoview of superimposed ribbon models of CusC (orange) and TolC (green; PDB-code 1EK9), viewed from the extracellular side (A) and from the side (B). The Cα r.m.s.d. between CusC and TolC is 1.61 Å. Panels (C) and (D) show the superposition of CusC and OprM (blue), the structures of which have a Cα r.m.s.d. of 1.35 Å.

**Figure 6:**
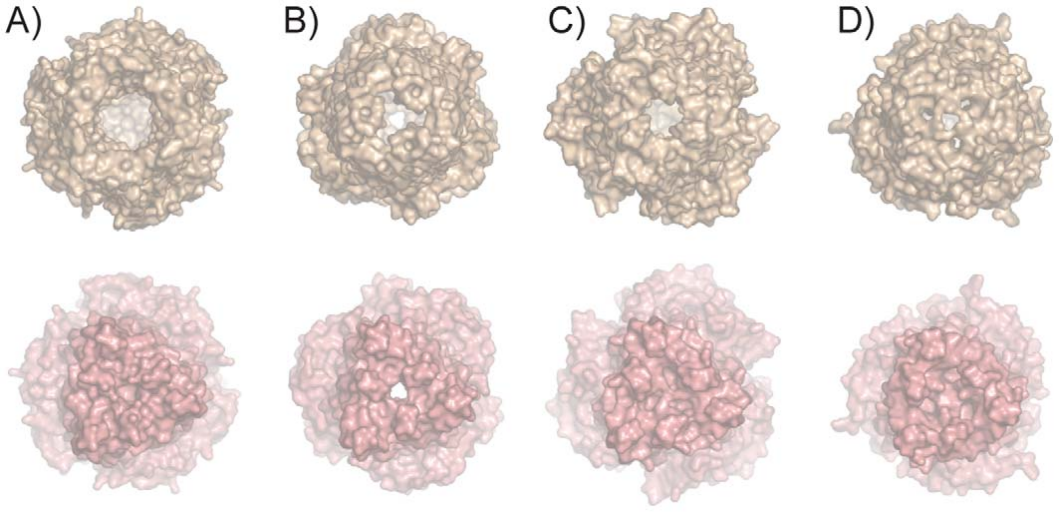
Structural comparisons of the intra- and extracellular openings of OMF proteins. Surface views from the extracellular side (top row) and from the periplasmic side (bottom row) of CusC (A), TolC (B), *P. aeruginosa* OprM (C; PDB-code 1WP1) and *Vibrio cholerae* VceC (D; PDB-code 1YC9).

TolC is not able to replace CusC with respect to conferring resistance to copper and silver ions *in vivo* [2]. The most likely reason for this observation would be an inability of TolC to form a functional pump with CusAB. From the model of the assembled AcrAB/TolC pump [12] it is clear that the outer membrane channel makes extensive interactions with the α-helical subdomain of the MFP. Since the structure of the α-helical subdomain of the MFP CusB is very different from that of all other known MFPs [15], it is likely that TolC will not be able to form a functional pump with CusAB. Moreover, the equatorial domains of CusC and TolC are quite different in structure (Fig. 5). Since these equatorial domains interact with the MFP this will also contribute to the inability of TolC to form a functional complex with CusAB. Alternatively, it is possible that TolC can form a functional pump by interacting with CusAB, but that specific structural features of CusC would confer specificity for monovalent cations (*e.g.* by having a narrow channel). The CusC structure shows that this possibility is unlikely. First, the α-barrels are very similar for CusC, TolC and OprM (Fig. 5), suggesting that the open channels in the assembled pumps may also be similar. In addition, despite the apparent specificity of CusC for small substrates [20], its extracellular opening is the largest observed for any OMF protein to date (∼30 Å diameter, measured as Cα-Cα distance; Fig. 6). By contrast, the extracellular openings of both TolC and OprM are much smaller (∼12 Å diameter) due to the inwardly-folded extracellular loops. Thus, it is clear that the CusC β-barrel does not present any selectivity filter or other barrier for diffusion of its substrates. The extracellular opening of CusC, with the loops splayed apart, is likely representative of a maximally open state of OMF proteins. The CusC α-barrel, by contrast, is closed towards the periplasmic end (Fig. 6), and will require substantial conformational changes to generate an open channel, similar to other efflux pumps [12].

The presence of the periplasmic copper binding protein CusF as part of the tetrapartite copper/silver efflux system has prompted the idea that the Cus system is not only involved in the extrusion of copper and silver from the cytoplasm, but also from the periplasm [17], [19]. For the latter, CusF has a possible role in the delivery of metal ions to CusB. In support of this notion, a direct and specific transfer of Cu(I) ions from CusF to CusB was recently reported *in vitro* [7]. In addition it was found that CusB has binding sites for Cu(I) and Ag(I) [15], the first evidence of a possible direct role of a membrane fusion protein in transport. From CusB, substrates are probably transferred to CusA and/or CusC. In our structure there are no openings in the CusC walls that would allow the acquisition of metals from CusB, but such passageways might be generated upon assembly of the intact efflux pump. It is unlikely that CusC has specific substrate binding sites, since soaking of Cu(I) and Ag(I) ions in the CusC crystals at high concentrations (10 mM) did not result in any observable binding. Moreover, the interior surface of a CusC monomer has only three methionine residues (nine for the trimer), all of which are far apart and do not form potential binding sites that are similar to the Cu(I) and Ag(I) ion binding sites observed in CusA, CusB and CusF. Thus, once the ions are in the CusC channel, transport will likely occur by diffusion together with the bulk solvent. In conclusion, CusC does not show structural features that would confer specificity towards metal ions, confirming phylogenetic analyses [4] and indicating that the narrow substrate specificity of the Cus pump most likely is caused by the inner membrane RND component CusA.

## Methods

The gene encoding for full-length *E. coli* CusC was amplified from DH5α cells (Invitrogen) by PCR and inserted into the pB22 expression vector [24] by ligation-independent cloning [25]. A hexa-histidine tag for metal-affinity purification was introduced after the C-terminal residue. The protein was over-expressed from *E.coli* C43 (DE3) cells via induction with 0.1% arabinose for 16–20 hrs at 20°C. Cells were ruptured by a single pass at 15,000–20,000 psi in a microfluidizer (Avestin Emulsiflex C-3), followed by membrane pelleting by ultracentrifugation. Inner membrane proteins were pre-extracted by 0.5% sarkosyl in 10 mM Hepes/50 mM NaCl pH 7.5 for 30 mins at room temperature, followed by centrifugation. The resulting pellet was extracted with 1% LDAO in 20 mM Tris/300 mM NaCl/10% glycerol pH 8 (TSB buffer) for 2 hrs at 4°C. After ultracentrifugation, the extract was loaded onto a 10 ml nickel column (chelating sepharose, GE healthcare). The column was washed with 15 volumes of TSB containing 0.2% LDAO and 20 mM imidazole, followed by elution with TSB containing 0.2% LDAO and 200 mM imidazole. CusC was further purified by gel filtration (Superdex-200) in 10 mM Na-acetate/50 mM NaCl/10% glycerol/0.05% dodecyl-β-maltoside (DDM) pH 5, followed by concentration (7–12 mg/ml; 50 kDa molecular weight cutoff) and overnight dialysis against the same buffer. The final yield of CusC was ∼3 mg per 12 liter of cells.

Hanging-drop crystallization trials were set up using in-house and commercial screens (MemGold; Molecular Dimensions) at 18 and 22°C. Small diamonds were obtained in MemGold 2/#23. These crystals were optimized in ∼35% PEG200/0.2 M (NH_4_)_2_SO4, 50 mM Na-acetate pH 4.0; they grew to maximum dimensions of ∼0.1×0.1×0.05 µm in two months. The crystals belong to space group R32 and have one molecule in the asymmetric unit (Vm = 3.6 Å^3^/Da, corresponding to ∼66% solvent). A native dataset was collected at NSLS beamline X6A on a crystal that diffracted to 2.2 Å resolution. Diffraction data were processed in HKL2000 [26]. The structure was solved by molecular replacement (MR) using Phaser [27] with *P. aeruginosa* OprM as the search model, since MR efforts using TolC were not successful. The likely reason for the MR failure using TolC likely lies in the low sequence identity to CusC (15%; compared to 45% sequence identity between CusC and OprM). Model building was done in COOT [28] and refinement with Phenix [29], to an R_work_/R_free_ of 19.8/24.3% (Table 1). The final model and structure factors have been deposited in the Protein Data Bank with accession code 3PIK.
